# Sweet taste sensitivity in pre-diabetics, diabetics and normoglycemic controls: a comparative cross sectional study

**DOI:** 10.1186/1472-6823-14-67

**Published:** 2014-08-13

**Authors:** Sudharshani Wasalathanthri, Priyadarshika Hettiarachchi, Shamini Prathapan

**Affiliations:** 1 Department of Physiology, University of Colombo, Colombo, Sri Lanka; 2 Department of Physiology, University of Sri Jayewardenepura, Gangodawila, Nugegoda, Sri Lanka; 3 Department of Community Medicine, University of Sri Jayewardenepura, Gangodawila, Nugegoda, Sri Lanka

**Keywords:** Pre-diabetes, Diabetes mellitus, Sweet taste, Detection threshold, Recognition threshold, Suprathreshold ratings

## Abstract

**Background:**

Increasing prevalence of pre-diabetes is an emerging public health problem. Decrease in sweet taste sensitivity which can lead to an increase in sugar intake might be a factor driving them to overt diabetes. The aim of the present study was to assess the sweet taste sensitivity in pre-diabetics in comparison with diabetics and with normoglycemic controls.

**Methods:**

Forty pre-diabetics, 40 diabetics and 34 normoglycemic controls were studied. The three groups were matched for age, sex and BMI. The division into groups was based on their glycated hemoglobin levels. The detection and recognition thresholds were determined by the multiple forced-choice method using sucrose solutions prepared in ¼ log dilutions. The intensities of perceived sensations for a series of suprathreshold concentrations of sucrose solutions prepared in ½ log dilution were determined by rating on a visual analogue scale. Statistical analyses were performed by SPSS version 21.

**Results:**

The mean (SD) detection thresholds of diabetic, pre-diabetic and normoglycemic groups were 0.025 (0.01), 0.018 (0.01) and 0.015 (0.01) respectively with a significant increase in diabetic group compared to normoglycemic group (p = 0.03). The mean recognition thresholds were not different among the three groups. When the intensity ratings for suprathreshold concentrations of sucrose were compared between the three groups, for all suprathreshold concentrations tested, significant differences were observed across the four concentrations (p < 0.001) and between groups in suprathreshold ratings (p < 0.05). Further analysis showed that the diabetic group had significantly lower suprathreshold ratings than the normoglycemic group (p < 0.001). Although all mean suprathreshold intensity ratings of the pre-diabetic group were between the normoglycemic and diabetic groups, the differences were not significant.

**Conclusions:**

This is the first study to demonstrate the sweet taste sensitivity in pre-diabetics. The findings of the present study do not support the hypothesis of decreased sweet taste sensitivity of pre-diabetics. However, the results confirm the previous findings of blunted taste response in diabetics. The observation of pre-diabetics having intermediate values for all taste thresholds and suprathreshold ratings warrants a future investigation with a larger pre-diabetic sample recruited with more specific screening criteria to test this hypothesis further.

## Background

Recent advancements in the food industry appear to have altered the food choices of people with food consumption driven more by pleasure than the nutritive value of foods [1]. Taste perception and food preferences are shown to be important determinants of dietary practices [2] which in turn contribute to the development of non-communicable diseases [3].

The sensation of taste is experienced when the chemical concentration of a tastant reaches a threshold level which activates taste receptors to generate action potentials in gustatory nerve fibers that are strong enough to elicit a taste perception [4]. Taste thresholds are modified by multiple factors including genetics [5], age [6], body weight [7], consumption of alcohol [8], smoking [9], acute and chronic diseases [10] and surgical interventions [11].

Impairment of taste sensation has been described long before in patients with diabetes mellitus (DM) [12,13] and the impairment is found to be mostly for the sweet sensation compared to other taste modalities [14,15]. Increase in taste thresholds is shown to be associated with hyperglycemia [16] with the presence of a significant correlation between the taste thresholds and the level of blood glucose concentration suggesting a blunted sweet taste response in patients with type 2 diabetes mellitus (T2DM) [14]. Since it is also observed that patients with T2DM crave for high carbohydrate containing foods [17], it is likely that these patients consume more sugar compared to non-diabetics. Even if there is no conclusive evidence to suggest whether the decrease in sweet taste sensitivity in T2DM patients occur as a result of an alteration of glucose homeostasis or vice versa, the blunted taste response is possible to facilitate a vicious cycle which leads to a deterioration of their glycemic control.

Although the association between DM and sweet taste sensitivity is investigated extensively, evidence on taste thresholds in pre-diabetics is lacking globally. As reviewed by [18] in 2012, 5 – 10% of pre-diabetic individuals become diabetic annually with an increasing prevalence of pre-diabetes worldwide. Life style adjustments which include dietary changes have been accepted as important strategies for stopping progression of pre-diabetes to diabetes [19]. Hence, knowledge about sweet taste sensitivity in pre-diabetics is worth exploring with a view to facilitate reverting a pre-diabetic to a normoglycemic via dietary interventions. Therefore, the aim of the present study was to assess the sweet taste sensitivity in pre-diabetics and to compare this with DM patients and with normoglycemic controls. This study focuses on pre-diabetics since appropriate timely intervention may be vital to stop or delay the progression of the pre-diabetic state to true diabetes.

## Methods

### Subjects

In this analytical cross sectional study, taste thresholds were compared in pre-diabetics with age, sex and body mass index (BMI) matched T2DM patients and with normoglycemic controls. Although a total of 191 subjects were studied, data of only 40 pre-diabetics, 40 diabetics and 34 normoglycemics were considered for analysis due to stringent matching of confounding baseline characteristics.

Patients with diagnosed T2DM aged between 20–60 years, attending the family practice centre of the University of Sri Jayewardenepura, Sri Lanka during a period of 4 months were included in the diabetic group. The employees in the same university with no history of diabetes were invited to be included in the pre-diabetic and control groups and were categorized into the two groups depending on their glycated haemoglobin (HbA1_C_) levels. HbA1_C_ levels were determined by the high-performance liquid chromatography method under strict quality assurance guidelines. Grouping of these subjects to pre-diabetics and normoglycemic controls was done with HbA1_C_ using the American Diabetes Association guidelines [20]. Individuals with mental and physical illnesses, those on medications affecting the smell and taste sensations, pregnant and lactating women and those with diseases of the oral cavity were excluded from the study.

#### Ethical considerations

This study was approved by the Ethics Review Committee of the University of Sri Jayewardenepura and informed written consent was obtained from participants prior to recruitment.

#### Data collection

Taste sensitivity testing was carried out in batches of 5–6 subjects per day. On the day of the tests, the participants were asked to arrive between 8 AM and 8:30 AM refraining from food, smoking, alcohol and betal chewing from 10 PM. the previous day to standardize the testing procedure with regard to the level of hunger/satiation [21]. Standard breakfast comprising of 3 slices of brown bread with margarine and a plantain was given 1 hour before sensitivity testing. An interviewer administered questionnaire was used to obtain demographic data, dietary history which included the details of sugar consumption of the subjects, the past medical history and details of all medications of the subjects. Measurements of height and weight were recorded. Blood (5 ml) was drawn to an EDTA tube for the estimation of HbA1_C_ and these values were used to identify pre-diabetic subjects from normoglycemic controls. Taste testing was performed in an odorless room and completed before 11 AM.

#### Threshold testing

Sucrose solutions which were prepared diluting sucrose in distilled water in successive dilutions of 1/4 log and 1/2 log steps were used for the estimation of detection and recognition thresholds and for supra threshold estimations respectively. The concentration gradients which were used for sucrose detection and recognition thresholds (1.25 × 10^3^ to 6.4 × 10^1^ mol/L) were based on previous literature [11] and confirmed by a pilot study done on 30 subjects. All taste thresholds were assessed by the same research assistant trained by the investigators.

Detection and recognition thresholds were determined by the multiple forced-choice presentation of freshly prepared sucrose solutions in order of ascending concentration starting from the lowest. The sucrose solution and distilled water were offered to subjects in 3 disposable cups in a pre-randomized order – two containing 10 ml distilled water and one containing 10 ml sucrose solution. They were asked to swish the solutions in the mouth for 5 seconds, spit out and pinpoint which cup contains the solution with a taste. The subjects were instructed to rinse the mouth with distilled water in between tasting the solutions to eliminate any remaining traces of sucrose in the mouth. In the event of giving an incorrect response or stating the inability to distinguish between the solution and distilled water, the subjects were presented with the next set of solutions which contained the sucrose solution with the next higher concentration of sucrose. Solutions were offered in this manner until the presence of a taste was identified correctly twice in succession. The concentration of the solution at which the participant was able to identify the presence of a taste first, was considered as the detection threshold and the concentration of the solution at which the participant was able to identify the quality of the taste first, was considered as the recognition threshold.

The perceived sensations of suprathreshold intensities of sucrose solutions presented randomly were determined by the ratings indicated by the subjects in a 230 mm visual analogue scale (VAS) graded from ‘0’ to ‘100’ which is a modification of the general Labeled Magnitude scale (gLMS) described in published literature [22]. The scale which is modified to suit our population was pre-tested in the pilot study. The top and bottom ends of the vertical scale had intensity labels with descriptive adjectives, “strongest imaginable” and “barely detectable” respectively, indicated in the native language (Sinhala) of the participants. Prior to introducing the test solutions of varying concentrations, each subject was allowed to taste the two solutions with the lowest (6.4 × 10^-3^ mol/L) and the highest (2.02 mol/L) concentrations for them to familiarize with the two ends of the scale. The 4 sucrose solutions (2.02 × 10^-2^, 6.40 × 10^-2^, 2.02 × 10^-1^ and 6.40 × 10^-1^ mol/L) were randomly presented to the subjects 1 minute apart. They were asked to taste each solution for 5 s, spit out and rate the intensity of it by marking a cross on the scale taking into account the intensities perceived for concentrations representing the ends of the scale. Instructions were given to rinse the mouth with distilled water in between tasting each sucrose solution. Since each concentration was rated 3 times, the average of these ratings was considered as the intensity rating for that particular concentration.

#### Statistical analysis

The sociodemographic factors were presented as counts for categorical variables and as means and standard deviations for continuous variables. As only 8% (n = 10) of the sample were smokers and also because the smokers were distributed in almost equal proportions amongst the three groups, smoking was not considered in subsequent analysis. ANOVA was performed and post hoc comparisons were made using the Tukey’s procedure to compare the differences between the three groups in detection and recognition thresholds and in the amount of sugar consumed per day.

Differences in Suprathreshold ratings were analyzed using ANOVA for repeated measures with the three groups (diabetics, pre-diabetics and normoglycemic controls) as between subject factor and suprathreshold intensity ratings as within subject factor. Application of this model showed positive kurtosis. Mauchly’s test indicated that the assumption of sphericity has been violated, therefore degrees of freedom were corrected using Greenhouse-Geisser estimates of sphericity (ϵ = 0.43). When ANOVA revealed significant effects, post hoc Tukey’s analysis was conducted. The criterion for statistical significance was at p < 0.05.

## Results and discussion

Taste thresholds for sucrose in pre-diabetics, diabetics and normoglycemic controls were determined in this study which is the first study reporting the taste sensitivity in pre-diabetics.

Baseline characteristics of the subjects in the three groups are shown in Table 1. Since the three groups were closely matched for age, sex and BMI, the influence of these confounders on taste thresholds were assumed to be negligible.

**Table 1:** Baseline characteristics of pre-diabetics, diabetics and normoglycemic controls

**Characteristics**	**Normoglycemic controls (n = 34)**	**Pre-diabetics (n = 40)**	**Diabetics (n = 40)**
Age (years)*	45.1 (8.9)	45.9 (9.4)	45.7 (8.4)
Sex (M/F)	11/23	17/23	22/18
BMI (kg/m^2^)*	23.4 (2.7)	25.4 (3.1)	24.7 (3.1)
HbA1_C_ (%)*	5.3 (0.2)	6.0 (0.2)	8.6 (2.1)

*Mean and (standard deviation).

The sugar consumption (number of teaspoons of sugar consumed per day) was significant across the groups, F (2, 113) = 10.2, p = 0.00. Tukey post-hoc comparisons of the three groups indicated that the diabetic group (*M* = 1.85, 95% CI [1.54 - 2.16]) had a significantly lower sugar consumption than the pre-diabetic group (*M* = 2.60, 95% CI [2.39 - 2.81]), *p* = 0.000, and the normoglycemic group (*M* = 2.53, 95% CI [2.28 - 2.77]), p = 0.001. However the causality cannot be established as this was not a follow up study. None of the subjects in this study reported to consume artificial sweeteners. Although carbohydrate craving is found to be associated with DM [17], it is surprising to find that diabetic patients in our study has consumed significantly lower amounts of sugar in their beverages when compared to the other groups. This finding may be attributed to the dietary advice these patients get form various awareness programs held routinely in the country. However, not considering the consumption of sugar in other sweet foods in this analysis can be considered as a limitation.

It is reported that more than 250 medications affect smell or taste [23]. Thus, we did not recruit subjects on any routine medications other than on hypoglycemic agents or antihypertensives. Subjects on medicines such as antibiotics taken for short durations were also excluded. Although taste disturbances are reported with metformin [24] and losartan [25,26], we were unable to exclude subjects on these medications due to practical reasons. Therefore, since our study sample had diabetics on metformin (n = 10) and losartan (n = 04), and pre-diabetics on losartan (n = 4), the influence of these drugs on our results cannot be excluded.

Detection and recognition thresholds of pre-diabetics, diabetics and normoglycemic controls are shown in Table 2. Although we hypothesized that Sri Lankans who consume a diet rich in spices to have lower taste sensitivity when compared to people consuming bland foods, the detection and recognition thresholds for sucrose in normoglycemic individuals were found to be comparable to studies done elsewhere [27-29] in populations with different dietary habits.

**Table 2:** Detection and recognition thresholds of pre-diabetics, diabetics and normoglycemic controls

	**Normoglycemic controls (n = 34)**	**Pre-diabetics (n = 40)**	**Diabetics (n = 40)**	**p value**	**Post hoc test (p value)**
	**Mean (SD)**	**Mean (SD)**	**Mean (SD)**		
Detection Threshold (mol/L)	0.015 (0.01)	0.018 (0.01)	0.025 (0.01)	0.04	0.03*
Recognition Threshold (mol/L)	0.043 (0.03)	0.044 (0.02)	0.054 (0.07)	0.35	-

SD – standard deviation.

*Normoglycemic controls were significantly different to diabetics.

The association between diabetes mellitus and sweet taste sensitivity was known for more than three decades [13]. In the present study, a significant increase in the mean detection threshold for sucrose was observed in diabetics compared to normoglycemics. This finding is in agreement with other studies where the sweet taste was elicited by either sucrose [16] similar to the present study, or by glucose [15]. However contrary to what was expected, this study failed to demonstrate significant differences in recognition thresholds among the three groups. Although Perros et al. [30] in a similar study showed significantly higher recognition thresholds in diabetics compared to non-diabetic controls, the mean HbA1_C_ level in the diabetic group was 12.6% indicative of very poor glycemic control in those patients. The mean (SD) HbA1_C_ in our diabetic group was 8.6% (2.1) indicating that our diabetic patients had a better glycemic control. This may be the reason for this difference, as significant correlation between taste thresholds and HbA1_C_ has been described in previous studies [14].

**Figure 1:**
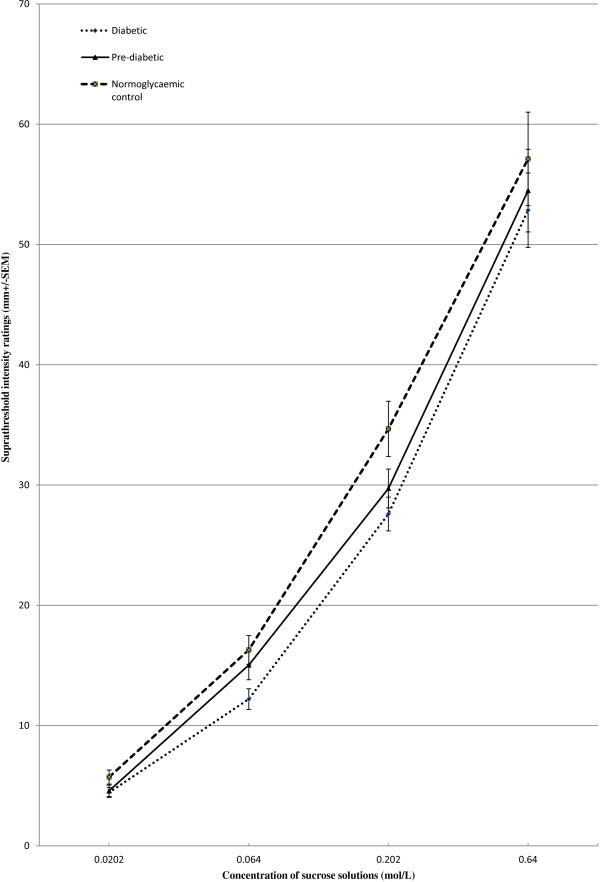
**Suprathreshold intensity ratings of pre-diabetics (n = 40), diabetics (n = 40) and normoglycemic controls (n = 34).** The intensities (mm) of perceived sensations for four suprathreshold concentrations (0.0202, 0.064, 0.202, 064 mol/L) of sucrose in pre-diabetics, diabetics and normoglycemic controls are shown. Tukey’s post hoc analysis confirmed that intensity ratings of diabetics were significantly lower (p < 0.001) compared to normoglycemic controls.

Suprathreshold intensity ratings are reported to be far superior than threshold estimations to assess the taste response in an individual [31]. The suprathreshold intensity ratings among the three groups are shown in Figure 1. There was a significant difference across the four suprathreshold concentrations, F(1.3, 131.9) = 671.2, p < 0.001 and significant differences between groups, F(2, 100) = 5.8, p < 0.05 in supra threshold ratings. There was also a significant interaction between the suprathreshold ratings and groups, F(2.6, 131.9) = 3.08, p < 0.05. Following up this interaction, the post hoc indicated that the diabetic group has significantly lower suprathreshold ratings on VAS when compared to the normoglycemic group (p < 0.001).

An important objective of this study was to explore the taste world of pre-diabetics. We expected pre-diabetics to perform worse on taste assessments compared to normoglycemics as taste impairments have been observed even in newly diagnosed diabetic patients [30]. Although the pre-diabetics in the group we studied seem to have lower suprathreshold ratings compared to normoglycemics for all concentrations studied, these differences were not statistically significant. Our findings may be explained by considering the factors which have significant correlations with taste thresholds such as blood glucose concentrations and HbA1_C_ levels [14], duration of diabetes and peripheral neuropathy [32], all of which are unlikely to have an impact on taste thresholds in pre-diabetics. However, this observation may also be attributed to the very close HbA1_C_ values between pre-diabetics (mean of 6.0%) and the normoglycemic controls (mean of 5.3%) in the present study. Furthermore, the findings of a very recent study [33] that has demonstrated a very low specificity of HbA1_C_ when compared to oral glucose tolerance test (OGTT) for identifying pre-diabetes in South Asians warrants a further investigation using OGTT as a screening test for pre-diabetes.

This study also confirms the previous findings of blunted taste response in diabetics. In a previous research it was shown that diabetics desire for high carbohydrate foods especially when they are in poor glycemic control [17]. Although we can suggest that this observation may be due to the altered sweet taste sensitivity in diabetics, according to our knowledge there is no reported data to confirm whether this desire actually drives the diabetics to use more sugar to improve the taste of their food.

Taste is an important sense evolved to drive food intake. Evidence of reversal of blunted sweet taste response with correction of hyperglycemia [34], decrease in sweet taste thresholds during weight loss [35] and modulation of taste thresholds by changing the concentrations of various neurotransmitters [36] indicate that the sweet taste thresholds are not static. Therefore, future research may be targeted towards new strategies to increase the sensitivity of sweet taste receptors in pre-diabetics and diabetics to obtain a desired sweet taste by consuming low concentrations of sugar. This may help in reverting pre-diabetics to normoglycemics and to improve the glycemic control of diabetics.

## Conclusions

This is the first study to demonstrate the sweet taste sensitivity in pre-diabetics. Results of this study do not support the hypothesis of decreased sweet taste sensitivity of pre-diabetics when compared to normoglycemic subjects. Although the detection and recognition thresholds, and the ratings for all suprathreshold concentrations of sucrose of pre-diabetics lie in between the values obtained by diabetics and by normoglycemic controls, none of the differences were statistically significant. A future study with a larger pre-diabetic sample recruited with more specific screening criteria may be useful to test this hypothesis further. However, the results of this study confirm the previous findings of blunted taste response in diabetics. Although future research should be aimed at increasing the sensitivity of taste receptors, the present knowledge regarding taste sensitivity may be used in dietary counseling to adjust the mindset of these patients to consume less sugar.

## Competing interests

The authors declare that they have no competing interests.

## Authors' contributions

SW and PH conceived the study, participated in the design and supervised taste threshold estimations, SP performed the statistical tests and analyzed the data. All authors (SW, PH and SP) participated in interpretation of data, drafting the manuscript and final revision of the manuscript. All authors read and approved the final manuscript.

## Pre-publication history

The pre-publication history for this paper can be accessed here:

http://www.biomedcentral.com/1472-6823/14/67/prepub
